# Ethylene is involved in pistil fate by modulating the onset of ovule senescence and the GA-mediated fruit set in *Arabidopsis*

**DOI:** 10.1186/1471-2229-11-84

**Published:** 2011-05-16

**Authors:** Pablo Carbonell-Bejerano, Cristina Urbez, Antonio Granell, Juan Carbonell, Miguel A Perez-Amador

**Affiliations:** 1 Centro Nacional de Biotecnología (CNB), Consejo Superior de Investigaciones Científicas (CSIC), Cantoblanco, 28049 Madrid, Spain; 2 Instituto de Biología Molecular y Celular de Plantas (IBMCP), Universidad Politécnica de Valencia (UPV)-Consejo Superior de Investigaciones Científicas (CSIC). Ciudad Politécnica de la Innovación (CPI), Ed. 8E, Ingeniero Fausto Elio s/n, 46022 Valencia, Spain

## Abstract

**Background:**

Ovule lifespan is an important factor in determining the ability to set fruits and produce seeds. Once ovule senescence is established, fruit set capacity in response to gibberellins (GAs) is lost. We aimed to elucidate whether ethylene plays a role in controlling ovule senescence and the fruit set response in *Arabidopsis*.

**Results:**

Ethylene response inhibitors, silver thiosulphate (STS) and 1-methylcyclopropene (1-MCP), were able to delay the loss of pistil response to GA_3_. In addition, ethylene insensitive mutants *ein2-5 *and *ein3-1 *showed delayed loss of pistil response, as in plants treated with STS and 1-MCP, while constitutive mutant *ctr1-1 *displayed premature loss of response. The analysis of the expression of ethylene biosynthesis genes suggests that ethylene is synthesised in ovules at the onset of ovule senescence, while a transcriptional meta-analysis also supports an activated ethylene-dependent senescence upon the establishment of ovule senescence. Finally, a *SAG12:GUS *reporter line proved useful to monitor ovule senescence and to directly demonstrate that ethylene specifically modulates ovule senescence.

**Conclusions:**

We have shown that ethylene is involved in both the control of the ovule lifespan and the determination of the pistil/fruit fate. Our data support a role of the ovule in modulating the GA response during fruit set in *Arabidopsis*. A possible mechanism that links the ethylene modulation of the ovule senescence and the GA_3_-induced fruit set response is discussed.

## Background

The pistil is a highly specialised floral organ designed to facilitate fertilisation, seed development and dispersal. Pistils become mature fruits by following a complex developmental programme triggered by ovule fertilisation, and by the hormonal signal cascade that follows. In the absence of this triggering event, the pistil's autonomous developmental programme leads to organ senescence after a few days [1-4].

Pistil senescence has been studied in pea (*Pisum sativum*) and *Arabidopsis *(*Arabidopsis thaliana*) plants. Unpollinated pea pistil senescence involves programmed cell death, which initiates at 2-3 days post-anthesis (DPA) [1,5,6]. Its onset correlates with both the expression of proteolytic activities [7-9] and the whole pistil's cell degradation [2], including DNA fragmentation in specific cells at both the ovary wall and ovules [6]. More recently, we showed that the development of the *Arabidopsis *unfertilised pistil differs from that of pea since the *Arabidopsis *ovary wall shows developmental characteristics that are shared with a developing fruit, while senescence is specifically established first at the stigma, and then progresses from basal to apical ovules [4].

One physiological marker of pistil senescence in both pea and *Arabidopsis *is the loss of the pistil's capacity to develop into a parthenocarpic fruit in response to exogenous gibberellic acid (GA_3_) [4,5]. The loss of pistil response to GA_3 _in *Arabidopsis *correlates with the onset of ovule senescence and its acropetal progression along the ovary [4]. In addition, several mutants with defects in ovule development showed a reduced fruit set response to GA_3 _[4]. Collectively, these data suggest that viable non-senescing ovules play a critical role in promoting fruit set in response to GA in *Arabidopsis *unfertilised pistils. The identification of the physiological and molecular factors regulating pistil/ovule senescence is important since the pistil's capacity to develop as a fruit is lost when senescence is initiated. Therefore by delaying ovule senescence, pistil longevity is expected to increase. This can lead to important biotechnological applications because reduced pistil longevity can be a limiting factor for sexual reproduction and fruit production [10-13].

Ethylene is involved in the control of several terminal processes during vegetative and reproductive development, including senescence of leaves [14-16], senescence and abscission of floral organs [3,17-19] and ripening of fruits [20]. In pea, ethylene regulates both petal and unfertilised whole pistil senescence [6,21]. Ethylene production increases during pea flower senescence, and the inhibition of ethylene action with silver thiosulphate (STS) delays senescence symptoms, including a postponed loss of the capacity to set parthenocarpic fruits in response to GA_3 _[6].

Ethylene signalling has been extensively reviewed in recent years [22-25]. Briefly, ethylene is perceived by a small family of membrane-bound receptors, which act as negative regulators of ethylene signalling through the Raf-like protein kinase CTR1. EIN2 is a positive regulator of ethylene response [26] and acts downstream of CTR1. The EIN3 and EIL1 components are transcription factors that act downstream of EIN2 and can activate ethylene responses.

This work aimed to characterise the ethylene involvement in the initiation and progression of *Arabidopsis *unpollinated pistil senescence by paying special attention to the potential effects of this hormone on ovule senescence and GA-induced fruit set response. Our data strongly suggest that ethylene modulates the onset of ovule senescence and, therefore, the time window for the GA-induced fruit set of pistils in *Arabidopsis*.

## Results

### Ethylene signalling modulates pistil responsiveness to GAs

**Figure 1:**
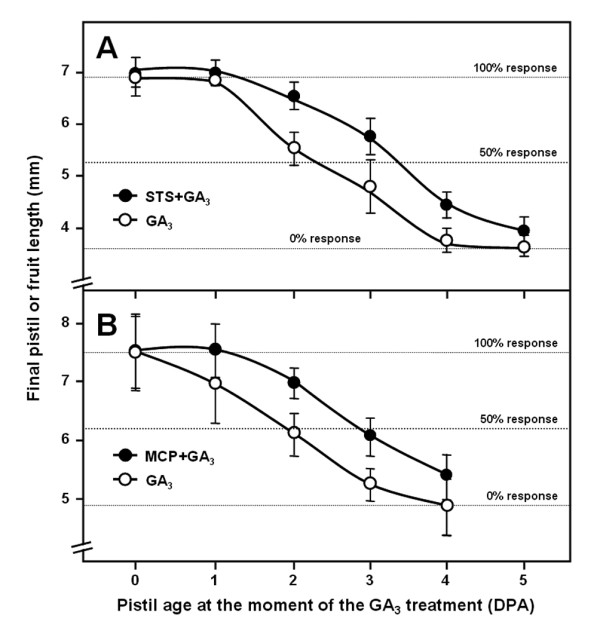
**Inhibition of ethylene perception delays loss of pistil fruit set responsiveness to GA**_**3**_. (A) GA response of STS-treated pistils. Pistil or fruit length in inflorescences treated with 50 μM STS at 5 and 3 days before GA_3 _treatment (●), and in control plants (**o**). (B) GA response of 1-MCP treated pistils. Pistil or fruit length in inflorescences treated daily with 1000 ppm 1-MCP from 1 day before anthesis to the day of GA_3 _treatment (●), and in control plants (**o**). Plants were in the *cer6-2 *background. Unfertilised pistils of different ages were simultaneously treated with 330 μM GA_3_. Pistil or fruit length was measured 10 days after GA_3 _treatment, and the data (mean ± SE) were plotted against the pistil age at the time of treatment. Experiments were repeated three times.

To test whether ethylene plays a role in pistil responsiveness to GAs, we first used two inhibitors of ethylene action, STS and 1-methylcyclopropene (1-MCP) to check if they affect the elongation triggered by GA_3 _when applied to unpollinated pistils. Inhibition of ethylene action postponed the loss of pistil fruit set responsiveness to GA_3 _by about 1 day (Figure 1). Both STS- and 1-MCP-treated pistils still maintained a 50% response at 3 DPA, which is the response shown by control untreated pistils at 2 DPA. On the other hand, the inhibitors did not affect the maximum length reached by parthenocarpic fruits. Therefore, the pharmacological approach indicates that ethylene plays a role in modulating the timing of pistil responsiveness to GAs and, thus, in pistil senescence.

**Figure 2:**
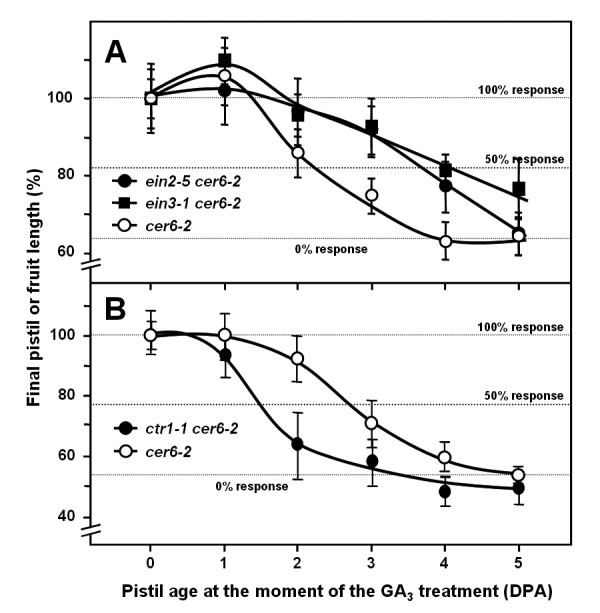
**Ethylene insensitive or constitutive response mutants show delayed and premature loss of pistil fruit set responsiveness to GA_**3**_**, respectively**. **(A) GA response in pistils of ethylene insensitive mutants *ein2-5 *(●), *ein3-1 *(■), and in control plants (**o**). (B) GA response in pistils of the ethylene constitutive response mutant *ctr1-1 *(●), and in control plants (**o**). Plants were in the *cer6-2 *background. Unfertilised pistils of different ages were simultaneously treated with 330 μM GA_3_. Pistil or fruit length was measured 10 days after treatment, and the data (mean ± SE) were plotted against the pistil age at the time of treatment. Data were normalised, and the size of the fruits treated at 0 DPA was 100%. Experiments were repeated three times.

Ethylene's implication in the control of pistil viability through the pistil fruit set response to GAs was further confirmed by a genetic approach. This involved testing pistil responsiveness to GA_3 _in ethylene-insensitive mutants *ein2-5 *and *ein3-1*, and in the ethylene constitutive response mutant *ctr1-1 *(Figure 2). Ethylene-insensitive mutants showed an approximately one-day delay in their loss of pistil responsiveness to GA_3_, a similar trend to that observed for the STS- and 1-MCP-treated pistils. Conversely, the loss of GA response in *ctr1-1 *took place one day earlier if compared to the control. These results genetically support ethylene's involvement in the modulation of pistil senescence.

Ethylene signalling mutations also affected pistil and fruit growth. In the completely insensitive *ein2-5 *mutant, pistils at anthesis were similar to those in parental plants, although the parthenocarpic fruits at 10 DPA after GA_3 _treatment were significantly larger (Additional file 1). On the other hand, constitutive *ctr1-1 *already displayed significantly shorter pistils at anthesis, and final fruit length was also significantly shorter than in parental plants.

### Activation of ethylene biosynthesis and response genes upon unfertilised ovule senescence

**Figure 3:**
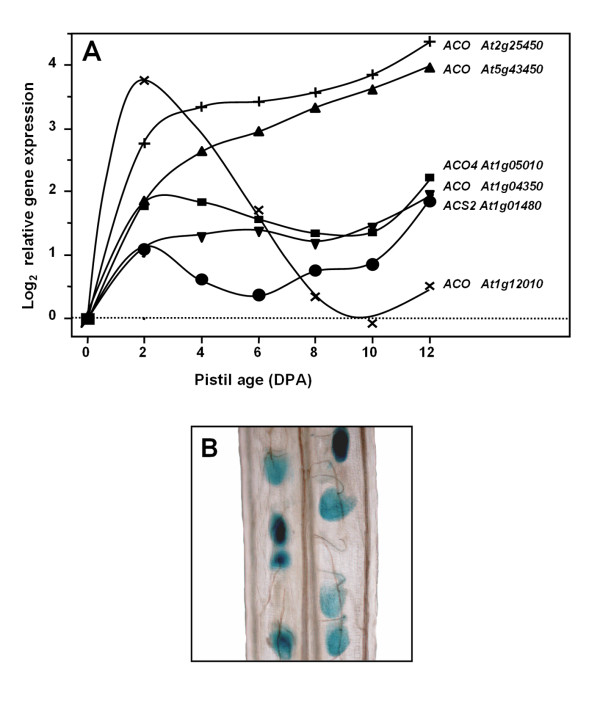
**Up-regulation of ethylene biosynthesis genes in ovules during unfertilised pistil development. **(A) Expression profile of genes of the ethylene biosynthesis differentially expressed during unfertilised pistil development. Data from the microarray analysis in [4] were used. *ACO*, ACC oxidase; *ACS*, ACC synthase. (B) *GUS *expression under the control of the *ACS2 *promoter in ovules at 2-3 DPA.

A transcriptomic analysis of *Arabidopsis *unfertilised pistils carried out previously [4] was revisited to indirectly test whether the ethylene biosynthesis pathway could be activated in unfertilised pistils. Several genes encoding ethylene biosynthesis enzymes, 1-aminocyclopropane-1-carboxylic acid (ACC) OXIDASES (ACOs) and ACO-like, were up-regulated at 2 DPA (Figure 3A). The expression of other genes of the ethylene biosynthesis was not detected or did not significantly change during unfertilised pistil development (data not shown). We studied how senescence affects the expression of ACC SYNTHASE (ACS), the enzyme catalysing the limiting step in ethylene biosynthesis, in unfertilised ovules by testing those transgenic lines that express GUS under the control of *ACS *promoters [27]. Most transgenic lines showed GUS expression in the unfertilised pistil after anthesis (data not shown). One interesting finding was that *ACS2 *[TAIR:*At1g01480*] was up-regulated in the unfertilised ovule; the GUS expression directed by the *ACS2 *promoter was detected at 2-3 DPA in the unfertilised pistil and was ovule-specific (Figure 3B). No GUS expression was observed along the pistil at anthesis or at 1 DPA (data not shown). It is remarkable to note that the temporal and spatial gene expression patterns of genes of the ethylene biosynthesis and GUS activity in the *ACS2:GUS *line closely matched the unfertilised ovule senescence [4].

In view of the fact that the up-regulation of those genes of the ethylene biosynthesis was coincident with the onset of ovule senescence, the ethylene transcriptional response was also analysed. To this end, we made use of previously published transcriptomic data for leaf senescence, which compared the wild-type and the *ein2 *ethylene-insensitive mutant [28], and also for the unfertilised pistil's post-anthesis development [4]. We identified those genes induced during leaf senescence in the wild-type, but not in the *ein2 *mutant (ein2/wt expression ratio below 0.5 [28]), and those up-regulated in the unfertilised pistil at 2 DPA (genes showing more than 2-fold change increase of expression between 0 and 2 DPA [4]) (Additional file 2). Of the 78 ethylene-dependent (EIN2-dependent) leaf senescence-induced genes [28], 75 were present in both microarray platforms used, of which 25 (33%) were up-regulated in 2 DPA unfertilised pistils (Additional file 2). This implied a significant enrichment in the EIN2-dependent leaf senescence-induced genes among those induced in 2 DPA unfertilised pistils (Table 1). On the other hand, a lower amount (21.7%) of the leaf senescence-induced genes identified by Buchanan-Wollaston *et al. *[28] was also up-regulated in the pistil at 2 DPA (Additional file 2). The significant enrichment in the senescence-activated genes dependent on ethylene among the up-regulated genes in the pistil at the onset of ovule senescence further suggests that ethylene plays a role in the process.

**Table 1:** Significant enrichment of genes induced during leaf senescence and EIN2-dependent leaf senescence among those induced in unfertilised pistils from 0 to 2 DPA

Term	Genes in platform	Positives in pistil	% genes in pistil	% genes in platform	Odds ratio	*p-*value
EIN2-dependent Leaf Senescence	75	25	3.4	0.3	2.65	4.6 E-18
Leaf Senescence	826	179	24.6	3.3	2.28	3.8 E-92

*p*-value: < 0.05 in a Fisher's exact test after Benjamini and Hochberg correction. Platform: gene set (20,560 genes) shared by Qiagen-Operon AROS [4] and Affymetrix ATH1 [28] microarrays. Odds ratio in Log_e_.

### The onset of ovule senescence in unfertilised pistils is affected in ethylene signalling mutants

The progression of ovule senescence along the pistil closely matches the loss of pistil growth responsiveness to GAs [4]. Here we show that ethylene modulates the initiation of the pistil's loss of GA response. In addition, the expression data also support the activation of ethylene biosynthesis and response upon the onset of ovule senescence. To directly test whether ovule senescence could be regulated by ethylene, we analysed the expression of the senescence marker gene *SAG12 *[TAIR:*At5g45890*] in wild-type and ethylene signalling mutant plants by using a line that expresses GUS under the control of the *SAG12 *promoter (*SAG12:GUS*) [29,30].

**Figure 4:**
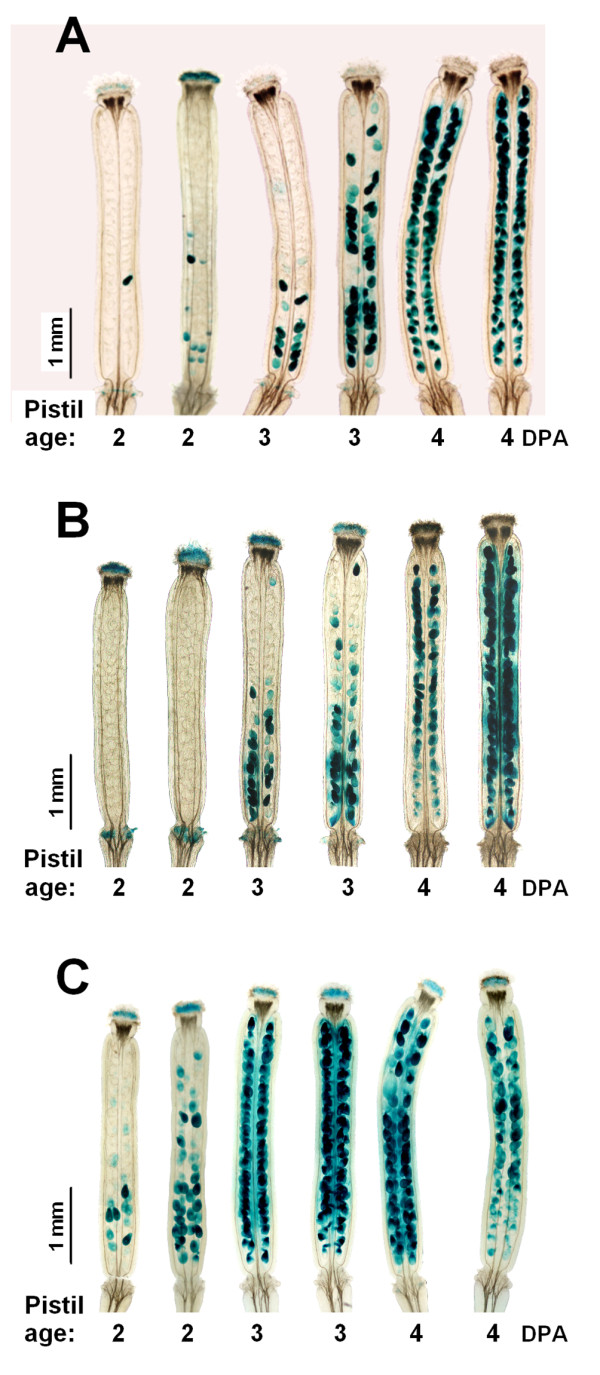
**GUS expression under the control of the *SAG12 *gene promoter during unfertilised pistil development in ethylene signalling mutants. **(A) GUS histochemical assay in unfertilised pistils of the *SAG12:GUS *line reveals onset of ovule senescence at 2 DPA, and that senescence progressed acropetally over the next two days. (B and C) GUS histochemical assay in the unfertilised pistils of the *SAG12:GUS *line in (B) *ein2-5*, and (C) *ctr1-1*. The onset of ovule senescence is delayed by 1 day in *ein2-5 *and is advanced by 1 day in *ctr1-1*. Senescence progression follows a similar kinetics as in the wild-type plant (A). Plants were in the *cer6-2 *background.

In a previous study, we demonstrated that the *SAG12 *expression was activated in unfertilised pistils shortly after anthesis, decreased afterwards, and increased again at the end of pistil development, at around 10-12 DPA. (Additional file 3). In the present study, by following the *GUS *expression under the control of the *SAG12 *gene promoter during unfertilised pistil development in Col-0 plants, we were able to confirm this expression pattern, which was coincidental with the senescence of ovules and valves, at 2 and 12 DPA, respectively (Figure 4A and Additional file 3 inset). GUS activity was first detected in the ovules at the basal zone of the ovary by the end of 2 DPA. Afterwards, it progressed acropetally along the ovary, and had extended to all the ovules by 4 DPA. Strong GUS activity was detected in all the tissues of pistils at 12 DPA (Additional file 3, inset). By looking in more detail, we noted that the GUS signal in the *SAG12:GUS *line indicated that ovule senescence began at the chalazal end, and that it later extended to cover the whole ovule (Additional file 4).

Next we tested the *SAG12:GUS *expression pattern in the unfertilised pistils of ethylene mutants *ein2-5 *and *ctr1-1*. Consistently with the loss of pistil response to GA_3 _(shown in Figure 3), the ethylene-insensitive mutant exhibited a one-day delay in the initiation of the ovule senescence (Figure 4B). The ovule senescence of the *ein2-5 *mutant initiated at 3 DPA, while it initiated at 2 DPA in parental plants (Figure 4A). Once it is initiated, the progression of ovule senescence in the ethylene-insensitive mutant followed a similar kinetics to that in parental plants. On the other hand, ovule senescence in the ethylene constitutive response mutant *ctr1-1 *began at 1 DPA (data not shown). The number of ovules expressing GUS under the control of the *SAG12 *promoter in *ctr1-1 *at 2 DPA was much higher than in parental plants (Figure 4C). The progression of ovule senescence in *ctr1-1 *was similar to that in parental plants, and like that observed for the *ein2-5 *mutant. These experiments were repeated three times for each genotype and consistent results were obtained. These results are in agreement with our data obtained using inhibitors of ethylene action, and reveal that the role of ethylene in accelerating the onset of ovule senescence without affecting the progression pattern.

## Discussion

The experiments described in this article unveil the role of the hormone ethylene in modulating the onset of ovule senescence in *Arabidopsis *and, therefore, the period at which the pistil is competent to set fruits upon GA_3 _treatment. In *Arabidopsis *and other plant species, ethylene is dispensable for vegetative or reproductive development under favourable conditions. However, the ethylene pathway can prove vital for plant plasticity to overcome stressing environmental conditions [31-34]. Therefore, the modulation of the ovule lifespan and pistil fate by ethylene may be important to ensure seed production under adverse conditions.

### Ethylene modulates pistil competence to develop fruits

Blocking ethylene perception extends the period in which the pistil is able to grow in response to exogenous GA in *Arabidopsis*, thus supporting similar results previously described for the unfertilised pea pistil [6]. This suggests that ethylene plays a key role in modulating the timing of pistil senescence in pea and *Arabidopsis *since the loss of pistil growth-responsiveness to GA in both species correlates with the onset of pistil senescence [4,5]. The delay of the loss of the pistil responsiveness to GAs by blocking the ethylene response, using both genetic mutants (*ein2-5 *and *ein3-1*) and pharmacological treatments (1-MCP and STS), further support the role of ethylene in modulating the fate of the unfertilised pistils in *Arabidopsis*. Additional support derives from the shortened period of pistil responsiveness to GAs in the *ctr1-1 *constitutive ethylene-response mutant. However, the lack of ethylene signalling in the *ein *mutants, or after inhibiting perception upon STS- and 1-MCP-treatment, delayed but did not prevent the loss of fruit set responsiveness to GA. Therefore, ethylene is not necessarily behind the loss of this capacity, but acts as a modulator of its initiation instead.

### Ethylene affects pistil and fruit size

In *Arabidopsis*, enhanced growth is the major distinctive characteristic between fruit and unfertilised pistil development [4]. The longer final length in both the GA-induced fruits and unfertilised pistils in the *ein2-5 *ethylene-insensitive mutant, as well as their smaller size in the *ctr1-1 *constitutive ethylene-response mutant, suggest that ethylene controls pistil and fruit growth. A similar control of adult rosette leaf size by ethylene has also been reported [35-37]. Given the fact that unfertilised pistils and GA-induced fruits grow almost exclusively by cell expansion after anthesis [38], one may consider that ethylene signalling reduces pistil and fruit length by reducing cell growth. Increased stabilisation of DELLA proteins, repressors of GA responses [39], promoted by ethylene signalling via CTR1 may be one of the causes of growth inhibition, which has already been proposed for roots [40].

### Ovule senescence and ethylene

Ethylene synthesis is regulated by developmental signals and other hormones, including GAs, and is enhanced by stresses, ageing and senescence [25]. Here we show an increase in the activity of ethylene biosynthesis genes in the ovules of unfertilised pistils. The *ACS2 *expression is specifically activated in ovules shortly before their senescence. The *ACS2 *expression has previously been linked with floral organ senescence [41]; similarly, a correlation between programmed cell death and increased ethylene levels during wounding and leaf senescence has been found [42]. In addition, the high expression of an ACC oxidase [TAIR:*At1g12010*], specifically in the ovules of 2 DPA unfertilised pistils [4], also supports activation of ethylene biosynthesis upon the initiation of senescence in unfertilized ovules.

Ethylene biosynthesis could be up-regulated as part of the ovule developmental programme (i.e., ovule ageing) to precipitate the progress of ovule senescence. Therefore, increased ethylene synthesis or perception would result in premature ovule senescence. Indeed, the accelerated onset of ovule senescence in the *ctr1-1 *mutant supports a causal relationship between increased ethylene signal and premature ovule senescence.

Although ethylene modulates the onset of ovule senescence, as indicated by the alteration of the *SAG12 *expression in the unfertilised ovules of ethylene signalling mutants, our data indicate that ethylene is not absolutely necessary for the progression of ovule senescence. A small time window of competence of ethylene has also been found; for instance, in *Alstroemeria *flower senescence and abscission [43], in contrast to other species like petunia, where suppressing ethylene action is able to delay flower senescence for longer periods [44-46]. The cases described for leaves are also similar to our results in *Arabidopsis *pistils: ethylene signalling also accelerates, but is not strictly necessary for senescence onset in *Arabidopsis *[14,47], tomato [17] and *Nicotiana sylvestris *[48]. An *EIN2-*dependent modulation of the expression of ageing-regulated factors triggering senescence in leaves has been recently defined [49], and a similar mechanism may operate in the ethylene signalling-dependent modulation of ovule longevity.

It is possible that the ethylene production rate in those ovules undergoing senescence increases under stress conditions. Indeed, the ethylene response is activated in pistils after a few hours of salt stress [50], while approximately three quarters of ovules die prior to fertilisation under stress conditions [51]. This mechanism could reallocate nutrients and energy from senescent ovules to vital sink organs like developing seeds.

### Integration of ethylene into the regulation of ovule senescence and pistil responsiveness to GA

The modulation of two temporally correlated processes by ethylene, progressive ovule senescence along the pistil and loss of the pistil fruit set response to GA, and their alterations observed in ethylene mutants, strongly indicate a causal relationship. In light of this, we recently showed that mutants defective in ovule development have impaired response to GA_3 _in the unfertilised pistils [4]. All in all, these data suggest that a viable ovule is required to accomplish adequate pistil response to GAs, and that ethylene plays a key role in regulating this response.

**Figure 5:**
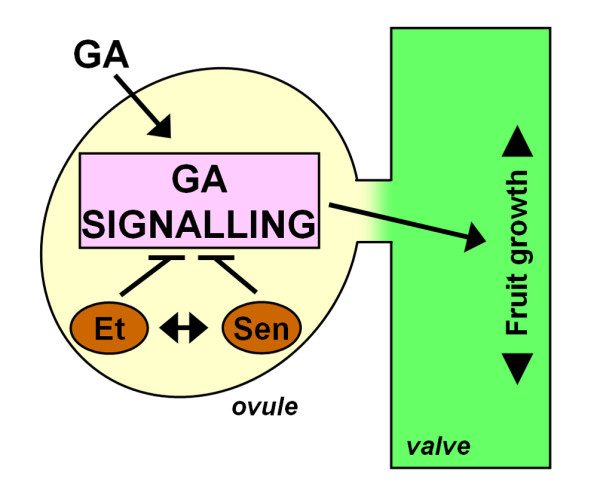
**Model for the ethylene regulation of ovule senescence and pistil response to GA_3_**. Proper ovules would be required to establish the fruit set response to GAs. Ethylene level would increase in ovules upon their senescence, accelerating the process and preventing the GA response. Ethylene can indirectly affect the GA response mechanism by promoting ovule senescence, or can also directly interfere with GA signalling (perception and/or response). GA, gibberellins; Et, ethylene; Sen, senescence.

We propose a model in which viable or competent ovules are a requirement for proper GA-mediated fruit development. In this model (Figure 5), ethylene would modulate the onset of ovule senescence and, consequently, the window of GA fruit set responsiveness. Therefore, the final parthenocarpic fruit length would depend on the number of viable ovules present in the pistil at the time of GA treatment given the correlation between the number of non-senescent ovules and the fruit size reached. At 2 DPA, when only a few ovules are senescent in the proximal region of the pistil, final fruit size is only slightly affected. Later at 3 DPA, most ovules are already senescent and fruit size reaches only partial length. Finally at 4-5 DPA, when all the ovules become senescent, the response is completely lost. In our model, we envision two different scenarios, depending on where GA perception and signalling are located: the ovules or the ovary wall. GA perception and/or signalling may be required in ovules to trigger fruit development, and the ethylene produced in ovules would directly prevent the response, for example, by the stabilisation of the DELLAs via CTR1 [40]. The limited fruit set response to GA_3 _shown by ovule defective mutants [4] supports this hypothesis.

Besides the direct effect of ethylene on GA signalling in the ovule, a different hypothesis can also be put forward. Ethylene could accelerate ovule senescence, which implies the degradation of all tissues and cell organisation which, in turn, would disassemble the GA perception and signalling machinery. In this case, the effect of ethylene would be indirect by promoting the degradation of all tissues in the ovule.

Despite all this evidence, the independence of the pistil responsiveness to GA of the ovule fate cannot be completely ruled out. Elucidation of the location of the relevant GA perception for fruit development, and the intercommunication between ovules and other ovary tissues, are essential to further define the model. However, the results obtained in the present work may be considered to extend ovule longevity using a biotechnological approach. For instance, expressing an ACC deaminase transgene or a dominant *etr1 *mutant allele under the promoter of a gene specifically activated early after anthesis in unfertilised ovules may serve to reduce ethylene production/signal and then delay ovule senescence.

## Conclusions

The data presented in our manuscript expands the physiological role of ethylene to modulate the onset of ovule senescence with new consequences for fruit set and development. Ethylene's involvement in ovule senescence further supports previous evidences suggesting that viable and non-senescing ovules are required to establish the parthenocarpic response in pistils. In addition, the present findings may be considered for biotechnological proposals; for instance, alterations in the ethylene signalling specifically directed in ovules could result in the prolongation of the ovule lifespan and, therefore, in greater seed and fruit yields.

## Methods

### Plant material

The *Arabidopsis thaliana *plants used were in the Col0 genetic background, except for 1-MCP and STS treatments, which were in L*er*. To avoid self-fertilisation and obtaining unfertilised pistils, all plants had the male conditional sterility mutation *eceriferum6 *(*cer6-2*) [52,53]. The *ACS2:GUS *line and *cer6-2 *in L*er *were obtained from the *Arabidopsis *Biological Resource Center (ABRC, http://www.biosci.ohio-state.edu). *cer6-2 *in Col0 was generously provided by Dr. A Vera (Universidad Miguel Hernandez, Spain). The *SAG12:GUS *transgenic line was a kind gift from Dr. RM Amasino (University of Winsconsin, WI, USA). *ein2-5, ein3-1*, and *ctr1-1 *were kindly provided by Dr. JM Alonso (North Carolina State University, NC, USA). *SAG12:GUS cer6-2, cer6-2 ein2-5, cer6-2 ein3-1, cer6-2 ctr1-1, SAG12:GUS cer6-2 ein2-5*, and *SAG12:GUS cer6-2 ctr1-1 *plants were generated by genetic cross. Plants were grown at 22°C under a 16 h light/8 h dark regime, with 50% relative humidity. To determine the age of each pistil in the primary inflorescence, the number and position of flowers at anthesis were recorded every day.

### Chemical treatments and fruit set responsiveness assays

Parthenocarpy was assayed by application of GA_3 _to unfertilised pistils. Inflorescences were sprayed with 330 μM GA_3 _(Fluka) and 0.01% (v/v) Tween 80, pH 7. Fruits and pistils were harvested 10 days after treatment, and scanned to measure final length with the ImageJ software [54].

STS and 1-MCP were used to inhibit ethylene action during the parthenocarpy responsiveness to GA_3 _assay. For STS, inflorescences were sprayed with 50 μM STS, 0.01% Tween 80 at 5 and 3 days before treatment with GA_3_. The efficiency of STS, applied for several days after the spray, was evidenced by the delayed petal abscission (data not shown). For each treatment, a fresh 20 mM stock of STS was prepared by mixing a 1:4 (v:v) ratio of 0.1 M AgNO_3 _(Sigma) and 0.1 M Na_2_SO_3 _(Sigma). Nearly all the silver in the solution was in the form of [Ag(S_2_O_3_)_2_]^3-^, which is the active complex for the inhibition of ethylene action. STS stock solutions were kept at 4°C in light-tight vessels.

For 1-MCP, pistils were treated daily from 1 day before anthesis to the day of GA_3 _treatment. Two hundred mg of a 1-MCP-releasing powder (SmartFreshTM, 0.14% of active ingredient; Rohm and Haas, Springhouse, PA, USA) was dissolved in 2.5 mL of water to provide a final gas concentration of 1000 ppm of 1-MCP inside a 0.125 m^3 ^air-tight glass box. Each day, three flowers at around 1 day before anthesis from 6-9 different primary inflorescences were emasculated to avoid self-fertilisation due to high humidity. Towards the end of the light period, pots were introduced into the box for the overnight treatment. Control plants were manipulated identically, but without 1-MCP.

### β-glucuronidase (GUS) histochemical assay

Samples were harvested and fixed for 30 min in ice-cold 90% acetone, washed once in the rinse buffer [50 mM NaPO_4 _buffer, pH 7.0, K_3_Fe(CN)_6_, K_4_Fe(CN)_6_, and 0.2% Triton X-100], and then vacuum-infiltrated and incubated for 24 h at 37°C in staining buffer (equal to the rinse buffer but supplemented with 2 mM X-GlcA (5-bromo-4-chloro-3-indolyl-b-D-glucuronide cyclohexylammonium) (Duchefa). K_3_Fe(CN)_6 _and K_4_Fe(CN)_6_, concentrations were adjusted for each line (2 mM for *SAG12:GUS *or 0.5 mM for *ACS2:GUS*). After staining, samples were dehydrated in a series consisting of 20, 35, 50, and 70% (v/v) ethanol. Finally, samples were cleared for 7 days in chloral hydrate prepared in a solution of chloral hydrate (Acros Organics, Geel, Belgium):glycerol:water in a 8:1:2 (g:mL:mL) ratio, and observed under an Eclipse E600 microscope.

### Transcriptional meta-analysis

The significant enrichment of leaf senescence-induced genes and EIN2-dependent leaf senescence-induced ones [28] was tested among those induced from 0 to 2 DPA in the unfertilised pistil [4]. For this purpose, only the gene set shared by Qiagen-Operon AROS [4] and Affymetrix ATH1 [28] microarrays (20,560 genes) was taken into account. Significance, according to a *p*-value below 0.05 in a Fisher's exact test after Benjamini and Hochberg correction, was analysed by mediating the Babelomics 4 functional enrichment tools [55].

The full microarray dataset from [4] is available in accession series in the NCBI GEO (Gene Expression Omnibus) repository [GEO:GSE13113].

## Abbreviations

ACC: 1-aminocyclopropane-1-carboxylic acid; ACO: ACC OXIDASE; ACS: ACC SYNTHASE; DPA: days post anthesis; GAs: gibberellins; GA_3_: gibberellic acid; GUS: β-glucuronidase; GEO: Gene Expression Omnibus; 1-MCP: 1-Methylcyclopropene; STS: silver thiosuphfate

## Authors' contributions

PCB generated the plant material and conducted most of the experiments. CU collaborated in the GUS assay experiments. AG and JC participated in the experimental design and edition of the manuscript. MAPA coordinated the study and drafted the manuscript. All the authors have collaborated in the edition of the manuscript and have approved it.

## Supplementary Material

Additional file 1 **Ethylene signalling affects pistil and fruit length**. Length of the untreated pistil at anthesis (left axis, in mm) and 10-day-old parthenocarpic fruit induced by GA_3 _treatment at anthesis (right axis, in mm) were measured in the control *cer6-2 *and ethylene response mutants *ein2-5, ein3-1*, and *ctr1-1*, all of them in the *cer6-2 *background. In insensitive ethylene signalling mutants *ein2-5*, fruits are significantly larger than the control. Conversely in the constitutive ethylene signalling mutant *ctr1-1*, both pistils and fruits are significantly shorter than in the control. Data are the mean ± SE. Two asterisks indicate significant differences (*p-*value < 0.01) with the corresponding *cer6-2 *control. Click here for file.

Additional file 2 **Comparative analysis of the transcriptomic data from unfertilised pistil senescence and EIN2-dependent leaf senescence**. The expression ratio of the genes up-regulated from 0 to 2 DPA in the unfertilised pistil according to [4] is shown. The *ein2*/wild-type expression ratio in leaves undergoing senescence from [28] is shown for those genes being also up-regulated during leaf senescence. Click here for file.

Additional file 3 ***SAG12 *expression during unfertilised pistil development**. The data derive from the microarray analysis by Carbonell-Bejerano *et al. *[4]. The SAG12 expression was statistically up-regulated in a biphasic fashion, with a prominent peak of expression at 2 DPA and a second one at 12 DPA. Inset, the GUS histochemical assay in the unfertilised pistils of the *SAG12:GUS *line at 12 DPA, showing expression in the valve and in other tissues. Click here for file.

Additional file 4 **Progression of ovule senescence monitored with the *SAG12 *expression in the unfertilised ovules of *SAG12:GUS *plants**. The *SAG12 *expression was first detected in ovules at 2 DPA and extended from outer integuments to inner layers. The expression finally extended to the chalazal pole by 3 DPA. The expression was never detected at the micropylar end. ch, chalaza; m, micropyle; i, ovule integuments; f, funiculus. Click here for file.
