# Supplementary figures and images for: Ethylene is involved in pistil fate by modulating the onset of ovule senescence and the GA-mediated fruit set in Arabidopsis
Source: BMC Plant Biol. 2011 May 16;11:84. doi: 10.1186/1471-2229-11-84 (PMC3124430; doi:10.1186/1471-2229-11-84)

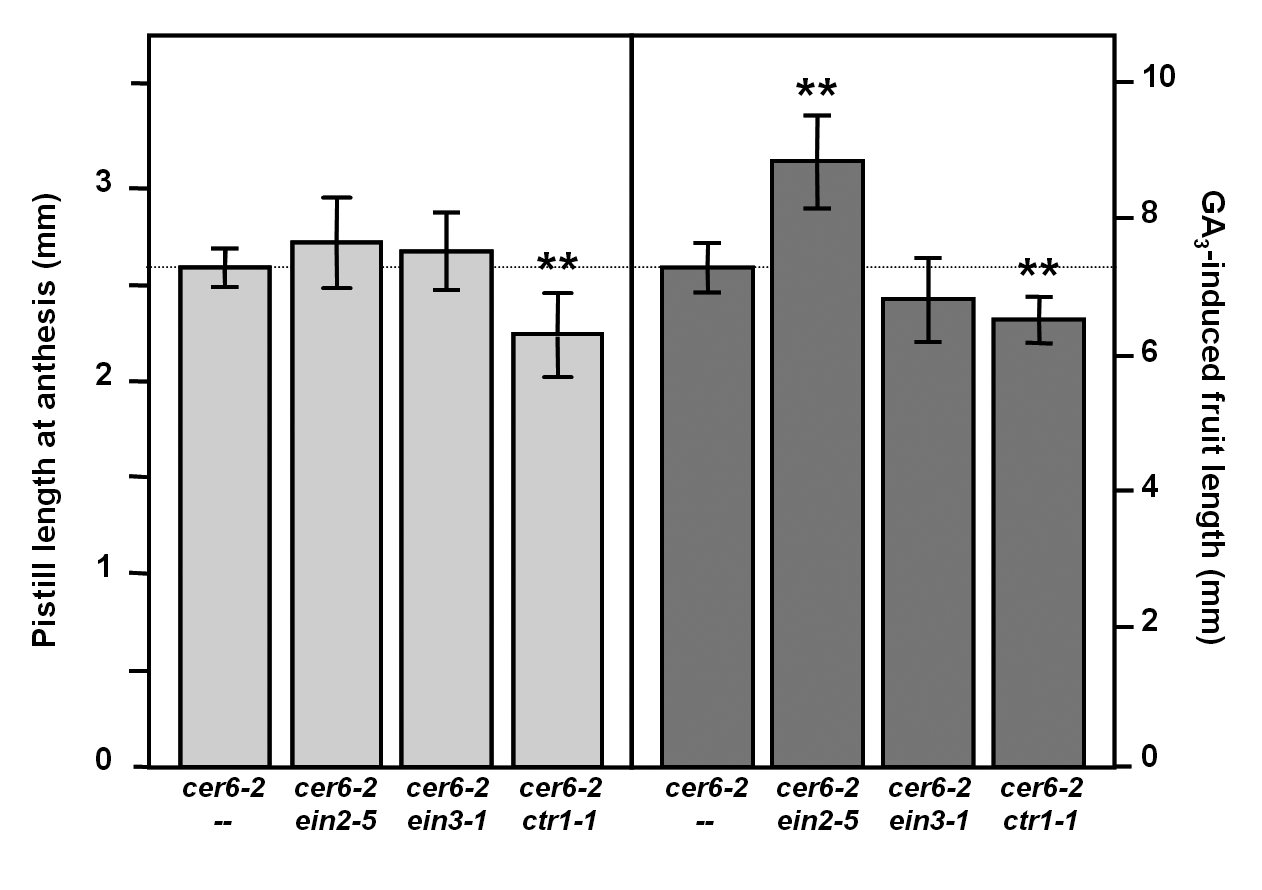

Supplement: Additional file 1 — Ethylene signalling affects pistil and fruit length. Length of the untreated pistil at anthesis (left axis, in mm) and 10-day-old parthenocarpic fruit induced by GA3 treatment at anthesis (right axis, in mm) were measured in the control cer6-2 and ethylene response mutants ein2-5, ein3-1, and ctr1-1, all of them in the cer6-2 background. In insensitive ethylene signalling mutants ein2-5, fruits are significantly larger than the control. Conversely in the constitutive ethylene signalling mutant ctr1-1, both pistils and fruits are significantly shorter than in the control. Data are the mean ± SE. Two asterisks indicate significant differences (p-value < 0.01) with the corresponding cer6-2 control. [file 1471-2229-11-84-S1.TIFF]

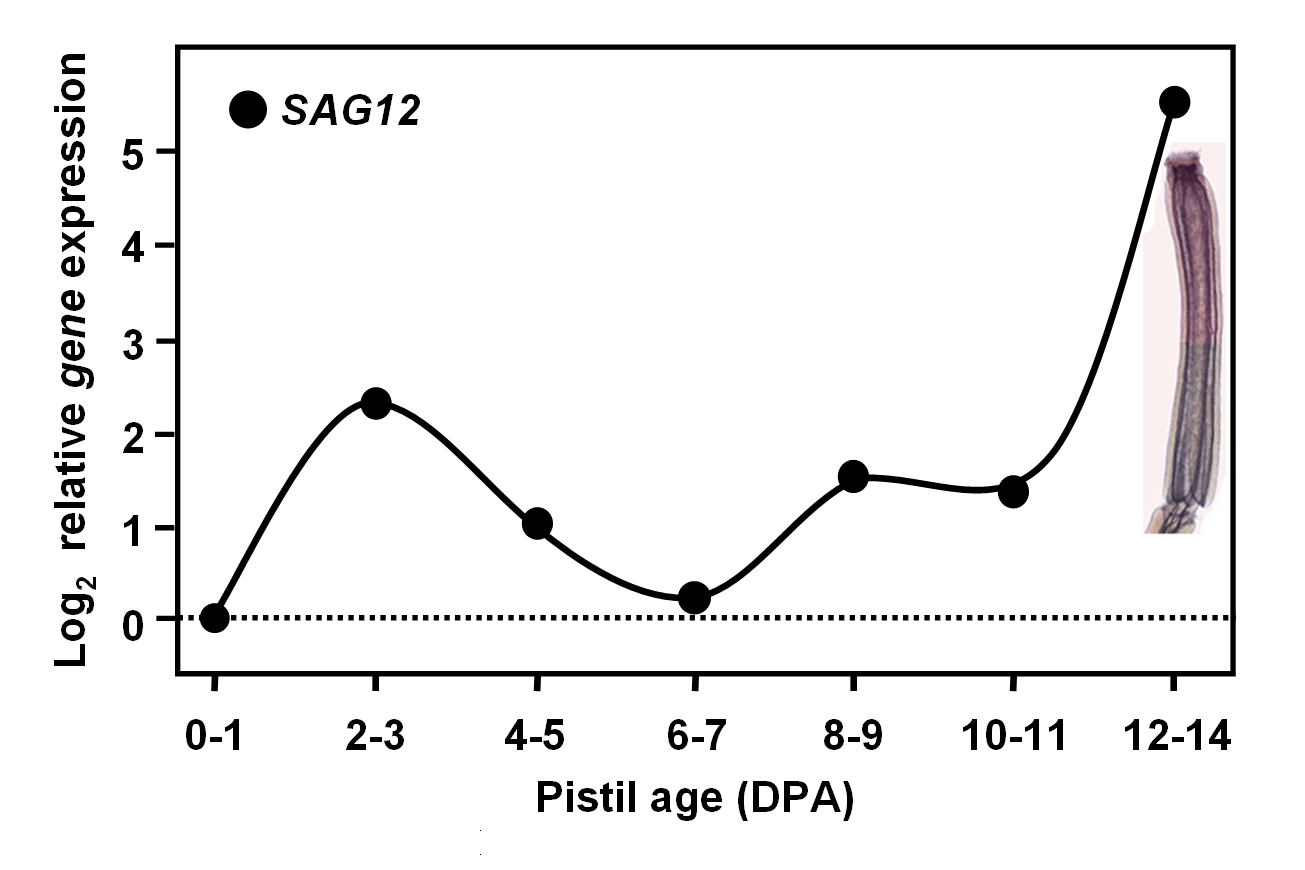

Supplement: Additional file 3 — SAG12 expression during unfertilised pistil development. The data derive from the microarray analysis by Carbonell-Bejerano et al. [4]. The SAG12 expression was statistically up-regulated in a biphasic fashion, with a prominent peak of expression at 2 DPA and a second one at 12 DPA. Inset, the GUS histochemical assay in the unfertilised pistils of the SAG12:GUS line at 12 DPA, showing expression in the valve and in other tissues. [file 1471-2229-11-84-S3.TIFF]

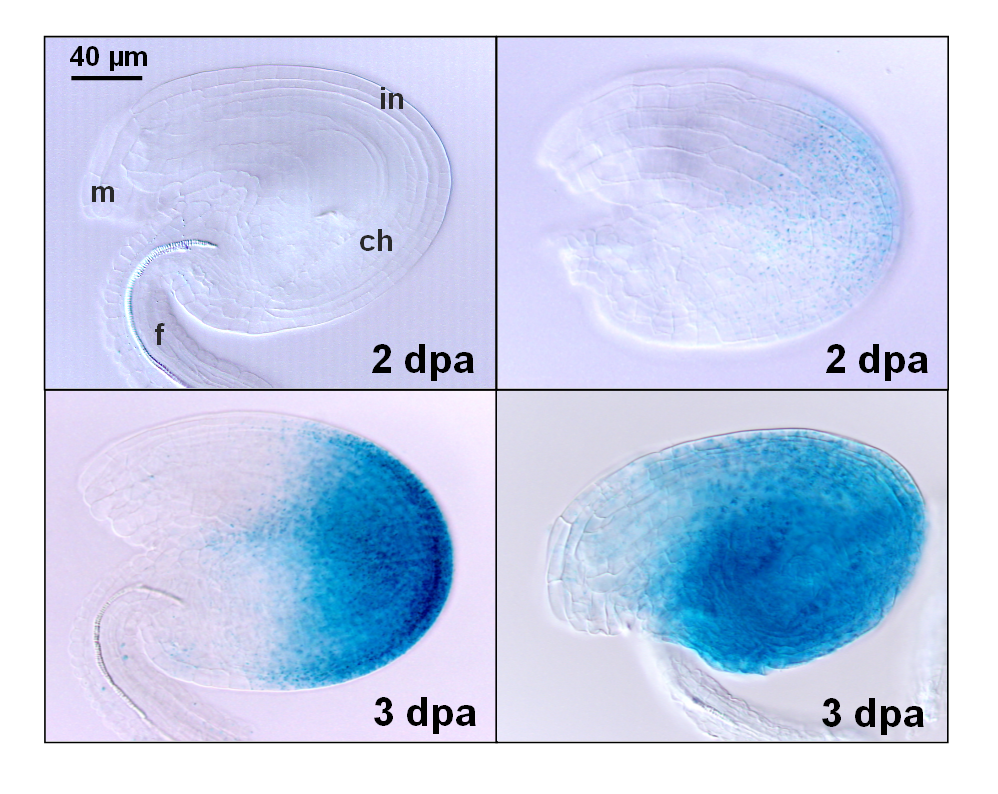

Supplement: Additional file 4 — Progression of ovule senescence monitored with the SAG12 expression in the unfertilised ovules of SAG12:GUS plants. The SAG12 expression was first detected in ovules at 2 DPA and extended from outer integuments to inner layers. The expression finally extended to the chalazal pole by 3 DPA. The expression was never detected at the micropylar end. ch, chalaza; m, micropyle; i, ovule integuments; f, funiculus. [file 1471-2229-11-84-S4.TIFF]
